# Incidence, reason for treatment delay and patient-reported outcome of patients affected by a chronic Achilles tendon rupture in a Swedish population

**DOI:** 10.1186/s12891-026-09890-y

**Published:** 2026-05-01

**Authors:** Niklas Nilsson, Elin Larsson, Emma Dyrehag, Michael Carmont, Annelie Brorsson, Katarina Nilsson Helander

**Affiliations:** 1 The Department of Orthopaedics, Institute of Clinical Science at Sahlgrenska Academy, Gothenburg University, Göteborgsvägen 31, Mölndal, Gothenburg, 431 80 Sweden; 2 Department of Orthopaedics, Sahlgrenska University Hospital, Mölndal, Sweden; 3 Department of Orthopaedic Surgery, Princess Royal Hospital, Shrewsbury and Telford Hospital NHS Trust, Shropshire, UK; 4 IFK Kliniken Rehab, Gothenburg, Sweden

**Keywords:** Achilles tendon rupture, Chronic Achilles tendon rupture, Surgical treatment, Non-surgical treatment

## Abstract

**Background:**

As the incidence of ATR is increasing the risk of ruptures being missed escalates and more injuries are suspected to become chronic. This study aimed to assess the incidence and causes of delayed diagnosis in chronic ATR. Additionally, the patient-reported outcome of surgical versus non-surgical treatment following delayed presentation was evaluated.

**Methods:**

The study is of patients with chronic ATR treated at the Sahlgrenska University Hospital between 2015 and 2020. Patients were identified using specific International Classification of Diseases (ICD) codes for ATR and included all patients in whom the treatment had been delayed for more than 14 days. The patients who accepted to participate in the study completed the Achilles tendon Total Rupture Score (ATRS) and a questionnaire regarding recovery rate in percentage.

**Results:**

Out of the 958 patients with ATR, 102 were identified as chronic, comprising 11% of the overall dataset. A total of 75 patients were included. Patients with chronic injury exhibited higher age, BMI, and comorbidity rates compared with patients with acute ruptures. Fifty-two (84%) patients delayed seeking medical attention. The rates of patients initially receiving an incorrect diagnosis was low, with 10 (1%) directly associated with trauma and 28 (3%) during later medical visits. Patients that were surgically treated (*n* = 57) for their chronic Achilles tendon rupture yielded significantly higher median (IQR) ATRS scores; 77 (50 ; 92) vs. 34 (23 ; 82) and recovery rates; 85% (70 ; 95) vs. 40% (20 ; 78) compared with patients treated with a non-surgical approach (*n* = 18).

**Conclusions:**

This study reveals that chronic ATRs constitute a significant portion of all ATR. These were primarily due to “patient’s delay” rather than the relatively rare misdiagnosis. Patient-reported outcomes, such as ATRS scores and self-reported recovery, exhibit considerable variability. Surgical intervention gave superior patient reported outcome compared with non-surgical treatment for patients affected by a chronic Achilles tendon rupture.

## Introduction

The majority of Achilles tendon ruptures are diagnosed during the first couple of days following injury when patients promptly seek medical attention [1]. These patients receive effective timely treatment, either surgical or non-surgical dependent on several factors. If more than 2–4 weeks elapse between the time of injury and diagnosis, or the provision of treatment, the rupture is termed chronic [2, 3]. Patients with chronic Achilles tendon rupture may experience symptoms such as pain, reduced strength in the lower leg, and difficulty pushing-off during walking, often significantly impacting their daily lives [3, 4]. The acute features of pain and swelling often subside during the first week. This is often mistaken for signs of minor injury healing, compounding a delay in presentation. Eventually, the loss of push off becomes more apparent, and patients may seek medical attention at later stages [5]. 

The delay in diagnosis stems from various factors. Patients may choose not to seek immediate medical care after the injury, hoping that the pain will dissipate, and normal function will return. Alternatively, healthcare professionals may misdiagnose the injury, providing patients with a reassuring, but incorrect assessment and thereby incorrect treatment. Studies indicate that up to 20% of acute ruptures go undetected and are often misdiagnosed as ankle sprains or injuries to the lower leg muscles [4, 6]. 

Given the increasing incidence of Achilles tendon ruptures in the population with increasing age, there is a growing concern about the potential rise in the number of chronic Achilles tendon ruptures. There is a lack of knowledge regarding the factors leading to late presentation following an Achilles tendon rupture and it is therefore important to investigate the factors associated with late presentation and diagnosis. The objective of this study was to determine the incidence and outcome of chronic Achilles tendon ruptures in a Swedish population.

## Method and materials

Ethical approval was granted by the Swedish Ethical Review Authority (DNR 2021 − 01779). The study is comprised from individuals with a diagnosis of chronic Achilles tendon rupture who received diagnosis and/or treatment at Sahlgrenska University Hospital between January 1, 2015, and December 31, 2020. Patients with an International Classification of Diseases (ICD) code S860 (indicating Achilles tendon injury) and M66.3 (indicating spontaneous rupture of the flexor tendon) were identified. To ascertain whether the patients had indeed been diagnosed with ATR, medical charts were reviewed, and those who had been diagnosed with an ATR were included in the cohort. Chronic Achilles tendon rupture was defined as a rupture diagnosed more than two weeks after the initial injury [3, 7]. Patients who received an appropriate diagnosis and treatment provision but did not heal, elongation, or those sustaining re-rupture, were excluded from the study group [8, 9]. All patients gave written informed consent for participation prior to enrolment in accordance with the Declaration of Helsinki.

Patient medical records were reviewed to ascertain the date of injury and the date of the initial medical consultation. The day on which the healthcare system either diagnosed the rupture or suspected a chronic Achilles tendon rupture and subsequently referred the patient to a specialized orthopaedic clinic for confirmation, was identified and termed the “day of diagnosis”.

The surgical techniques mainly included gastrocnemius aponeurosis free flap as described by Nilsson Helander et al. [10] or a semitendinosus autograft as described by Nilsson et al. [11] but also included V-Y tendon plasty and flexor hallucis longus tendon transfer. The surgical technique, post-operative immobilisation or rehabilitation were not standardised. The patients that were treated non-surgically received treatment with orthosis and physiotherapy.

All eligible patients were invited to participate in the study by receiving a letter with questionnaires and a written consent of participation. Participants were asked to estimate their limitations and functional ability using the Achilles tendon Total Rupture Score (ATRS) [12] and additional questions including, if translated from Swedish to English: *“How would you describe your recovery after your Achilles tendon rupture as a percentage from 0-100? 0 stands for not recovered at all and 100 for fully recovered”*. These invitations were dispatched when at least one year following the date of surgical repair or the start of non-surgical treatment.

### Statistical analysis

Descriptive analyses were outlined using means and standard deviations (SD) for variables with normal distribution, while median and interquartile range (IQR) were utilized for non-parametric variables. Categorical variables were expressed as numbers and percentages. Differences between surgical and non-surgical treatment as well as chronic versus acute Achilles tendon ruptures were evaluated using a non-parametric Mann-Whitney U test. There was no sample size calculation performed as all available patients in the clinic were invited to participate.

## Results

### Patient characteristics

**Fig. 1:**
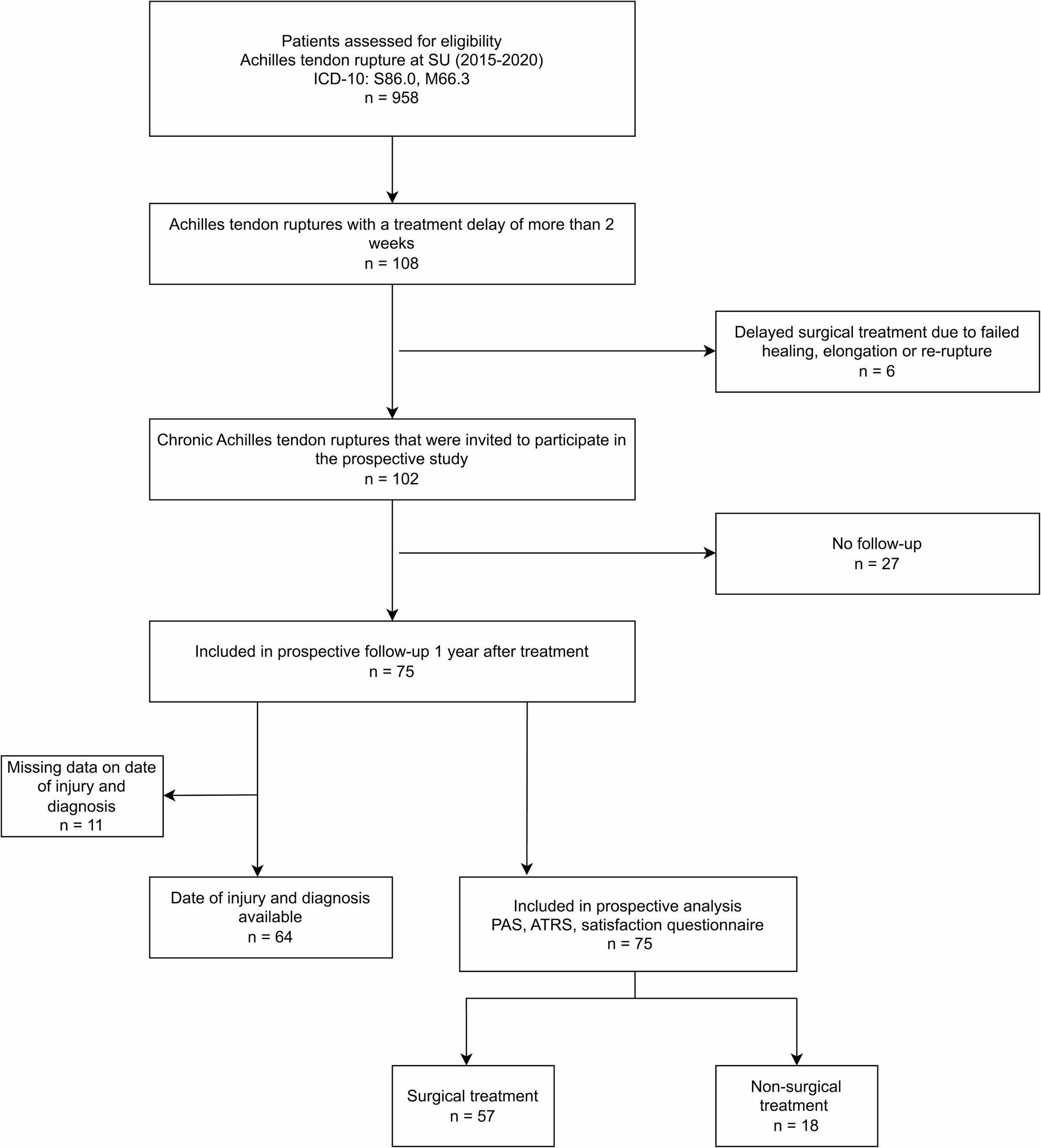
Flowchart describing the inclusion process for the study

A total of 102 (11%) patients from a larger study group of 958 patients with Achilles tendon ruptures were found to be chronic. Out of these 102 patients, a total of 75 participated in the study. The patient demographics are summarized in Table 1 and the inclusion process is described in the flowchart (Fig. 1). The main characteristics of these patients with chronic Achilles tendon ruptures were higher mean age, 64 vs. 47 (*p* < 0.001) and higher BMI, 27.4 vs. 26.2 (*p* = 0.02) compared to patients who had sustained an acute Achilles tendon rupture during the same period (*n* = 856). There was a non-significant difference in distribution of males and females in the two groups with 19.4% females in the acute group and 25.5% in the chronic group. The patients that were treated for a chronic Achilles tendon rupture were more often managed surgically compared to patients treated for an acute Achilles tendon rupture, 73% vs. 27% (*p* < 0.001).

Most patients were diagnosed in the emergency rooms or in primary care and were eligible for either non-surgical or surgical treatment. The surgical treatment consisted of Achilles tendon reconstruction using a free gastrocnemius aponeurosis flap (*n* = 41), a semitendinosus autograft (*n* = 8) or another surgical technique (*n* = 8), including Z tendon plasty, V-Y tendon plasty or flexor hallucis longus graft. Patients that received non-surgical treatment were those with low functional demands or potentially a high risk for surgical complications.

**Table 1:** The demographics for patients included, containing age, sex, BMI, side, treatment, previous tendinopathy, medications, and medical site for diagnosis

Demographic variable	
Age, mean (SD)	65 (12.7)
Sex, *n* (%)	
Female	19 (25)
Male	56 (75)
BMI, *n* (%)	
Normal (BMI < 24,9)	14 (21)
Overweight (BMI 25–29,9)	43 (63)
Obese (BMI > 30)	11 (16)
Side, *n* (%)	
Right	29 (39)
Left	45 (60)
Bilateral	1 (1)
Treatment, *n* (%)	
Surgery	57 (76)
Non-surgery	18 (24)
Surgical technique, *n* (%)	
Gastrocnemius free flap	41 (55)
Semitendinosus autograft	8 (11)
Other surgical technique	8 (11)
Non-surgery	18 (24)
Previous tendinopathy, *n* (%)	
Yes	25 (33)
No	50 (67)
Medications, *n* (%)	
Cortisone	6 (8)
Statins	8 (11)
Others	44 (59)
No medication	28 (37)
Location of Diagnosis, *n* (%)	
Emergency room	21 (28)
Orthopaedic clinic	14 (19)
Primary care	32 (42)
Others	8 (11)

*BMI *Body Mass Index

### Reasons for treatment delay

**Fig. 2:**
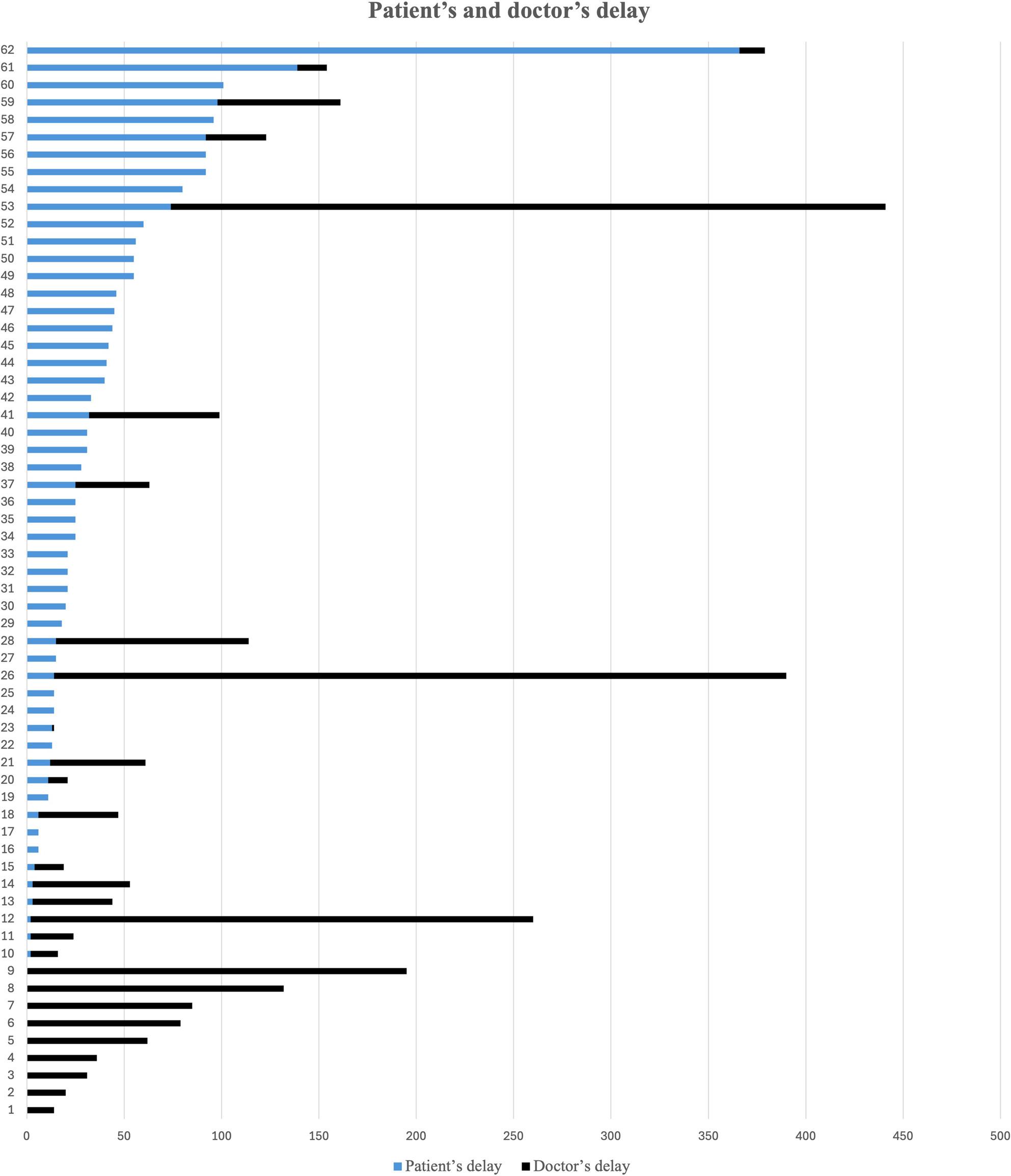
Figure describing time from injury until first clinical visit “patient’s delay”; blue, and time from first clinical visit until diagnosis “doctor’s delay”; black, for each individual patient. The time unit is in days

From the study group of 75 patients, the dates of injury, first medical visit and diagnosis could be determined in 62 patients (Fig. 2). A total of 10 patients sought medical care at the time of the injury and were misdiagnosed as a sprained ankle or other less severe musculoskeletal conditions - “doctor’s delay”, while 52 of the patients delayed before seeking medical attention - “patient’s delay”. In 34 (55%) of the these 62 patients, the injury was correctly diagnosed at the first medical visit and the rest (45%) was initially misdiagnosed. Only 16 (26%) out of 62 patients had not yet received their diagnosis 90 days post injury. When looking at the whole dataset 10 out of 958 (1%) were misdiagnosed in direct association to the trauma (doctor’s delay) while 28 out of 958 (3%) were misdiagnosed when they first attended medical help at a later stage (both doctor’s and patient’s delay).

### Surgical versus non-surgical treatment

The treatment modalities for chronic Achilles tendon ruptures were categorized into surgical or non-surgical interventions. The specific surgical treatments administered at Sahlgrenska University Hospital included free gastrocnemius aponeurosis flap (*n* = 41), a semitendinosus autograft (*n* = 8) or another surgical technique (*n* = 8), including Z-tendon plasty, V-Y tendon plasty or flexor hallucis longus graft.

Table 2 presents patient-reported recovery outcomes measured using the Achilles tendon Total Rupture Score (ATRS), with results presented in median values and interquartile ranges (IQR). In the surgery group, the median ATRS score was significantly higher compared with the non-surgery group (*p* = 0.006). Similarly, the number of recoveries displayed significant differences between the two treatment groups (*p* < 0.001).

**Table 2:** Patient-reported recovery regarding surgical and non-surgical treatment for patients with chronic Achilles tendon ruptures, measured in ATRS and percent, presented in median and interquartile range

	Surgery *n* = 57	Non-surgery *n* = 18	*p*-value
ATRS, median (interquatile range)	77 (50 ; 92)	34 (23 ; 82)	0.006
Recovery (%), median (interquatile range)	85 (70 ; 95)	40 (20 ; 78)	< 0.001

*ATRS *Achilles tendon Total Rupture Score

Patients were subsequently asked about their post-rupture physical activity levels compared to their pre-rupture status, with responses summarized in Table 3, a total of three patients failed to answer these questions and were consequently excluded. In the surgery group (n = 55), 29 patients, more than half of the cohort, reported either the same or increased activity levels following rupture, in comparison with their pre-rupture status. Conversely, in the non-surgical group (n = 17), only one patient reported maintaining the same activity level post-rupture, while the remaining 16 patients indicated a decrease in activity.

**Table 3:** Patient self-survey measurement of current activity compared to pre-injury level for patients treated surgically or non-surgically for a chronic Achilles tendon rupture

Current activity level compared to before rupture	Surgery *n* = 55	Non-surgery *n* = 17
Much more active	2	0
Somewhat more active	3	0
Same	24	1
Somewhat less active	19	8
Much less active	7	8

## Discussion

The most important finding in the present study is that chronic Achilles tendon ruptures account for approximately 10% of the whole population of Achilles tendon rupture patients giving a ratio of one chronic for every 9 acute ruptures. However, less than half of these chronic Achilles ruptures are caused by misdiagnosis or “doctor’s delay” which indicates that the frequency of missed Achilles tendon ruptures is as low as 1–3%. The present study also reaffirmed the commonly observed average age of patients diagnosed with chronic Achilles tendon is higher compared with those with acute ruptures. It is interesting to note that previous studies indicated a more equal gender distribution among patients with chronic Achilles tendon ruptures, however this study resembled the typical pattern observed in patients with acute ruptures. To our knowledge, this is the largest published clinical series of patients with chronic Achilles tendon ruptures and involves patients managed both surgically and non-surgically.

The decision between surgical or non-surgical treatment is highly individualized, considering the specific rupture characteristics, the patient’s overall health, previous activity, and future expectations. Surgical techniques and rehabilitation are often adapted based on the patient’s condition, leading to heterogenous patient cohorts [13]. Conversely, non-surgical treatment is typically offered to patients who are unsuitable for surgery or unable to complete the required rehabilitation programme. This calls for a risk of selection bias and needs to be taken into consideration when the results of this study are assessed. While many patients may face inherent challenges in achieving satisfactory recovery, the present study unequivocally demonstrates the superior recovery with surgical intervention. The lack of pre-operative data and a heterogenous patient group do however make it difficult to quantify the actual improvement through surgery. Previous studies evaluating acute Achilles tendon ruptures have demonstrated similar outcomes for surgical and non-surgical treatment [14] However, data on surgical versus non-surgical treatment for chronic Achilles tendon ruptures remain relatively unexplored and the risk of selection bias is high. Most studies consist of limited size case series of patients treated with the same surgical technique with good recovery and a high post-operative ATRS of 80–95 which is higher than presented in this study with a median ATRS value of 77 [15]. 

In a previous study, Winson et al. [16]. assessed the functional and patient-reported outcomes of 19 individuals with chronic Achilles tendon ruptures who underwent rehabilitation using the Swansea Morriston Achilles Rupture Treatment protocol (SMART) [17]. Over a span of 6.6 years, the mean ATRS score for the patient cohort rose significantly from 64.7 to 83 points. In terms of the present study, the patients that were treated using a non-surgical approach with rehabilitation, different walking-aids and ankle-foot-orthosis only reached a considerably lower median post-operative ATRS values of 34 points. In Winson et al.’s [16] study, the initial ATRS score was 64.7 points, which is relatively high when compared with other studies where surgical treatment is being evaluated [16]. This suggests that patients with such high scores before initiating treatment might be particularly well-suited for non-surgical treatment approaches, such as a structured rehabilitation protocol. In the present study, at least some patients who were treated non-surgically would probably have benefited by surgical reconstruction but were in-eligible due to comorbidities leading potential complications.

It must be appreciated that part of the period from which patients were recruited involved the period of the Covid pandemic 2020. During this time there were changes in sports participation with changes in the incidence of Achilles tendon ruptures. Patients were discouraged from visiting their family doctors and emergency departments in addition to increasing virtual consultants [18]. 

This retrospective study is subject to recall bias as data was collected at least one year after the reported rupture and solely are based on patient-reported outcomes. Consequently, the determination of recovery relies on subjective assessments, resulting in less reliable results. The heterogeneous nature of patients with chronic Achilles tendon ruptures compared with those with acute ruptures makes it harder to draw firm conclusions. Variability in age, co-morbidities, surgical techniques, rehabilitation protocol and medical history leads to diverse pain and function experiences. Additionally, data collection from medical records posed challenges, as not all information was consistently available. This resulted in data loss complicating conclusions in an already limited patient pool. The study had a substantial non-response rate (27%), potentially impacting the gender and age distribution. Despite these limitations, as well as the previously mentioned selection bias and lack of pre-operative data, the present study provides a unique and large compilation of patient data, combining information from medical records and self-reported patient data on risk factors and recovery.

## Conclusion

The present study shows that chronic Achilles tendon ruptures form a substantial proportion of all Achilles tendon ruptures. The primary cause for this is predominantly due to a delay in the patient’s presentation to medical practitioners rather than the uncommon misdiagnosis. The outcome shows a large variation in patient-reported outcomes, including ATRS and self-reported recovery. Surgical treatment results in superior patient reported outcome scores compared with non-surgical treatment following a chronic Achilles tendon rupture.

## Data Availability

The datasets used and/or analysed during the current study are available from the corresponding author on reasonable request.
